# A nutraceutical formulation combined with sclerofoam-assisted laser treatment ameliorates chronic venous insufficiency

**DOI:** 10.1007/s10103-022-03549-5

**Published:** 2022-04-18

**Authors:** Beniamino Palmieri, Maria Vadalà, Simone Ugo Urso, Laura Ornella Baldini, Caterina Fanelli, Julio Cesar Morales-Medina, Tommaso Iannitti

**Affiliations:** 1 Department of General Surgery and Surgical Specialties, University of Modena and Reggio Emilia, Modena, Italy; 2 Network del Secondo Parere, Via Ciro Bisi 125, 41124 Modena, Italy; 3 Phlebology Center, Medical Center 109, Bologna, Italy; 4 Phlebology Center, Il Faro Center, Melegnano, Italy; 5 Centro de Investigación en Reproducción Animal, CINVESTAV-Universidad Autónoma de Tlaxcala, AP 62, 90000 Tlaxcala, CP Mexico; 6 Southampton, UK

**Keywords:** Chronic venous insufficiency, Laser, Pain, Swelling, Itch

## Abstract

Chronic venous insufficiency has a high impact on the healthcare system due to its high incidence worldwide. We performed a study in 30 women with thigh and leg varices due to major saphenous vein valve incontinence with saphenous trunk reflux causing phlebo-lymphoedema to assess the efficacy of sclerofoam-assisted laser treatment combined with nutraceutical administration. The patients underwent endovascular combination sealing of the saphenous trunk with sclerofoam-assisted laser treatment technique into the major saphenous veins under low-volume tumescent anesthesia followed by intraoperative phlebectomies. Post-operatively, the patients received capsules containing *Aesculus Hippocastanum*, chondroitin sulphate, proanthocyanidins from *Pinus pinaster Aiton*, proanthocyanidins from *Vitis vinifera* L., hydrolysed marine collagen and carcinine dihydrochloride for 3 weeks. We evaluated the extracellular fluid volume of the lower limbs using bioimpedance spectroscopy pre- (T0) and post-surgery (T2) (impedance is a vector which is composed of two components, resistance [RES] and reactance [REA)]). In addition, we evaluated the following parameters pre- and post-surgery: pain, heaviness, paresthesia, itching, swelling, daily urine volume output and leg volume. Limb volume was significantly decreased at T2 compared to T0 (*p* < 0.01). RES and REA were significantly increased at T2 compared to T0 (*p* < 0.0001 and *p* < 0.01, respectively). A significant improvement in heaviness, paresthesia, pain, swelling and itch was also observed (all *p* < 0.0001) while no changes in terms of diuresis occurred. No adverse effects were observed. The present study shows a promising approach to the treatment of chronic venous insufficiency that warrants further clinical studies in larger cohorts of patients.

## Introduction

Chronic venous insufficiency (CVI) is a common condition with high impact on the healthcare system [1]. An epidemiological survey of 6009 patients, conducted in Belgium and Luxembourg, evidenced a high prevalence of CVI (61.3%) [2]. Prevalence will increase due to its main risk factors, including age, family history, obesity and necessity to stand at work [3]. For example, in a study including 541 Japanese women, 42% of subjects with varicose veins reported a positive family history compared with just 14% of women without disease; this difference is reduced with increasing age [4]. In the USA, the number of venous thromboembolism cases has been projected to increase from 1 million in 2010 to 1.8 million in 2050 [5], while varicose vein procedures are projected to increase by over 60% in the USA and Europe between 2013 and 2021 [6]. The CVI diagnosis is confirmed and classified with the Clinical, Etiologic, Anatomical, and Pathophysiological (CEAP) assessment (range: C0–C6) [7]. The CEAP classification for chronic venous disorders was developed in 1994 by an international ad hoc committee of the American Venous Forum, endorsed by the Society for Vascular Surgery, and incorporated into “Reporting Standards in Venous Disease” in 1995 [8]. Chemical endocavitary thermal treatments are a standard therapy due to low invasiveness, good efficacy and low complication rate [9]. Furthermore, they allow the patient to return to daily activities in a short time-frame post-treatment since CVI is cause of discomfort, pain, loss of working days, disability and reduction in quality of life [10–12]. The sclerofoam-assisted laser treatment (SFALT) technique 13 is routinely used in Italy and combines intravenous sclerofoam injection (polidocanol 3% or sodium tetradecyl sulphate 3% according to Tessari’s method) followed by endothelium stripping with fibre optic (600µ)–coupled laser diode (1470 nm wavelength) delivered at 40 J/cm fluence [14, 15]. This instrumental treatment can be combined to pharmacological or nutraceutical therapy to reduce pain, edema, inflammation and fluid retention (common side effects of traditional varicose vein treatments) and to induce rapid functional restoration in CVI patients [16]. Other add-on therapies can include (1) leg elevation [1, 17], (2) pharmacological therapy using vasoactive drugs that include coumarins, flavonoids, saponosides and other plant extracts, whose action is to improve venous tone and capillary permeability [1, 17] and (3) exercise therapy to improve calf muscle function [18]. The emergent role of herbal therapy administered orally or topically to patients has also been highlighted [19–21]. The advantage of herbal formulations is that multiple active principles are administered to the patient simultaneously, allowing synergistic action [22]. For this reason, the nutraceutical market in phlebology has sharply increased in the last 10 years [23]. Hence, in the current study, we evaluated the efficacy of SFALT combined with formulated compounds that display specific effects on the microcirculation of the operated leg.

## Materials and methods

### Patients

Thirty women with a mean age of 54.6 ± 1.4 years (mean ± standard error of the mean [SEM]) spontaneously appealing to our Network del Secondo Parere were voluntarily included in this study as they required a therapeutic approach to CVI treatment. The Network del Secondo Parere is a consultation referral web and medical office system using a panel of specialists, to whom any patient affected by any disease or syndrome and not adequately satisfied by his current diagnosis or therapy can apply to seek further medical advice [24]. Due to the physician–patient communication gap, several patients look for answers to their health problems on the Internet (“Web Babel Syndrome”). To deal with the problem of obsessive research by patients and resolve their health conditions, the Second Opinion Medical Consulting Network aims to be a useful problem-solving support system aimed to provide treatments and prognoses, preventing unnecessary investigations and unhelpful and expensive medical and surgical interventions [25–28].

### Informed consent and approval

All patients signed the informed consent and the study protocol was approved by the internal review board at the Network del Secondo Parere. The study was performed in agreement with the Helsinki declaration.

### Inclusion and exclusion criteria

All patients displayed thigh and leg varices due to major saphenous vein valve incontinence with saphenous trunk reflux causing phlebo-lymphoedema. We evaluated pain, heaviness, paresthesia, itching, swelling and daily urine volume output using a visual analogue scale (VAS; 0–10 cm). Leg volume was also determined using the mathematical formula of truncated cone [29]. Exclusion criteria were pregnancy, cancer, deep superficial vein thrombosis, obesity and cardiac, liver and kidney insufficiency.

### Treatment

Our protocol consisted in endovascular sealing of the saphenous trunk with the SFALT technique into the major saphenous veins under low-volume (0.2 ml) tumescent anesthesia followed by intraoperative phlebectomies. This technique is performed by simply puncturing the affected vein through the skin, without making any cuts. Under ultrasound guidance, the bar-tip 600 µm-diameter laser fibre (1470 nm wavelength, Biolitec AG, Germany) is introduced into the diseased vein using a power of 40 Joule/cm and positioned 1–2 cm below the vein, shrinking it a single cm at 200 J/cm. Keeping the fibre blocked, a 5 cc sclerofoam formulation (Varisolve®, BTG International, USA; 1:4 drug/air ratio administered using two 5-ml silicone-free syringes) is injected through the same laser introducer. The consequent spasm allows a subsequent laser-mediated shrinkage by means of a significantly reduced fluence. After removing the fibre, closure of the affected vein was confirmed by ultrasound imaging using a logic book XP system (GE Health-care, Buckinghamshire, UK).

After SFALT treatment, the patients wore elastic stockings day and night for 1 week, and only during the day-time for the following 3 weeks. Since the first post-operative day, each patient received 2 capsules/day of a galenic formulation containing 200 mg *Aesculus Hippocastanum*, 125 mg chondroitin sulphate, 60 mg proanthocyanidins from *Pinus pinaster Aiton*, 15 mg proanthocyanidins from *Vitis vinifera* L.,12 mg hydrolysed marine collagen and 10 mg carcinine dihydrochloride for 3 weeks. Symptoms were evaluated using the VAS scale and the extracellular fluid volume of the lower limbs was measured using bioimpedance spectroscopy (BIS) pre- and post-surgery [30]. This technique measures the electrical impedance of biological tissue through an electric field generated by alternate currents (maximum 200 µA) in multi frequency ranks from 3 to 1000 kHz. This impedance is strictly related to the tissue condition and it is a vector, which is composed of two components, resistance (RES), which is the expression of the edema, and reactance (REA), which expresses the tissue composition, both measured in Ohms. RES expresses extracellular liquids and is inverse to their concentration; REA is the result of the interaction between cell membranes and micro current delivery. The experimental outline consisted in patients undergoing clinical symptoms examination and evaluation via Doppler ultrasound, edema severity assessment using VAS, leg circumferential measurements using BIS in only 10 patients with chronic phlebo-lymphedema, and nutraceutical administration as above. Assessments were performed at baseline (T0), and at 60 days after treatment (T2).

### Statistical analysis

Statistical analysis was performed using GraphPad Prism 7 (GraphPad Software Inc., San Diego, CA, USA). Data was analysed using an unpaired *t*-test with Welch’s correction. *p* < 0.05 was considered significant.

## Results

**Fig. 1:**
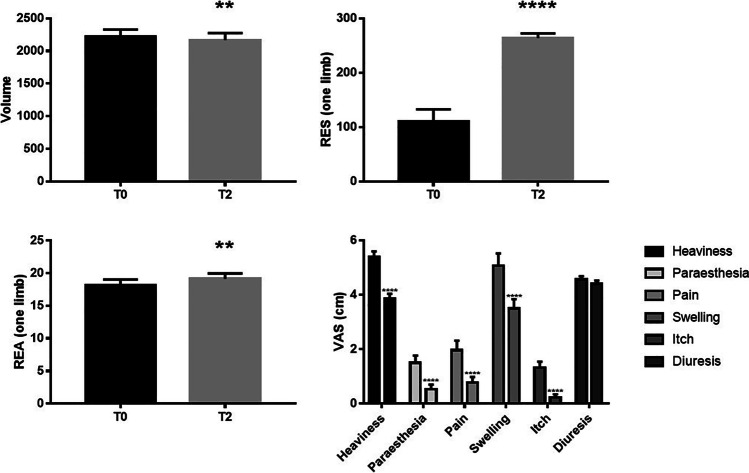
**A** Volume, **B** RES, **C** REA and **D** VAS of heaviness, paraesthesia, pain, swelling, itch and diuresis in CVI patients at baseline and post-SFALT and nutraceutical combination therapy

All the patients complied with the treatment schedule without showing any adverse effects. Only 4 patients required the use of painkillers (acetaminophen or ibuprofen) in the first post-operative day. Limb volume measured by BIS was significantly decreased at T2 compared to baseline (*p* < 0.01) (Fig. 1A). Similarly, RES and REA measured by BIS were significantly increased at T2 compared to T0 (*p* < 0.0001 and *p* < 0.01, respectively) (Fig. 1B and C). A significant improvement in heaviness, paresthesia, pain, swelling and itch was observed (all *p* < 0.0001) while no changes in terms of diuresis were reported, as assessed by Doppler ultrasound, VAS and medical evaluation (Fig. 1D).

## Discussion and conclusions

CVI has been treated with endovenous laser and radiofrequency in the last decade [15, 31]. However, these techniques are invasive and require multiple high-volume injections of tumescent anesthesia, which can lead to adverse effects, including post-operative pain, perivenous ecchymosis and hematoma in the treated areas, leading to patient’s dissatisfaction [32–35]. On the other hand, the SFALT method is an innovative procedure that can overcome the limitations of conventional treatments [36]. The low temperature and the sclerofoam injection allows a great saphenous vein tract reduction, avoiding the use of high-volume tumescent anesthesia, typical of traditional procedures.

No clinical study beyond case reports comparing this SFALT technique to surgery has been published yet. However, clinical case reports indicate that 80 to 90% of saphenous trunks remain occluded after 3 years when treated using the SFALT method [36]. Furthermore, this technique includes other benefits such as optimal cosmetic results and cost-effectiveness. There is no downtime and patients can resume normal activity immediately. However, this technique should be avoided in patients with severe thrombophilia and carried out with prophylaxis in patients with less severe thrombophilia. In regard to the nutraceutical formulation that we employed in this study, extracts from the seed of the horse chestnut have traditionally been used to alleviate several symptoms including lower leg swelling. These effects are largely due to an inhibitory effect on the catalytic breakdown of capillary wall proteoglycans [37]. Several clinical trials in CVI patients and in patients affected by varicose veins demonstrated the effectiveness of *Aesculus hippocastanum* extracts through the objective measure of reduction in lower leg edema and the subjective alleviation of leg pain, heaviness and itching [38–40]. In addition, *Pinus pinaster Aiton* administered as herbal supplement has been reported to have vasorelaxant activity, angiotensin-converting enzyme inhibiting activity, and the property to enhance the microcirculation by increasing capillary permeability [41]. Several clinical trials using red vine leaves (*Vitis vinifera*) confirmed improvement of microcirculation and oxygen supply, significant reduction of oedema and relevant improvement of leg pain, heaviness and tension [42–44]. In addition, chondroitin sulphate or chondroitin-4-sulphate is a natural glycosaminoglycan which possesses anticoagulant and antithrombotic effects [45].

Furthermore, in the present preliminary trial, SFALT combined with nutraceutical administration improved the patients’ quality of life and allowed a faster return to work and routine activities. Our study has limitations, i.e. limited number of cases included, the absence of control groups receiving only nutraceutical or SFALT intervention and lack of a blinding approach. Nevertheless, this proof-of-concept study has shown that this approach has no side effects and gives a promising outcome. Further studies taking into consideration the above-mentioned limitations will be conducted in the future.
